# A new potential mode of cardiorenal protection of *KLOTHO* gene variability in type 1 diabetic adolescents

**DOI:** 10.1007/s00109-020-01918-7

**Published:** 2020-05-20

**Authors:** Bartosz Słomiński, Monika Ryba-Stanisławowska, Maria Skrzypkowska, Magdalena Gabig-Cimińska, Małgorzata Myśliwiec

**Affiliations:** 1 Department of Medical Immunology, Faculty of Medicine, Medical University of Gdańsk, Dębinki 1, 80-211 Gdańsk, Poland; 2 Department of Medical Biology and Genetics, University of Gdańsk, Wita Stwosza 59, 80-308 Gdańsk, Poland; 3 Institute of Biochemistry and Biophysics, Polish Academy of Sciences, Laboratory of Molecular Biology, Kładki 24, 80-822 Gdańsk, Poland; 4 Chair and Clinics of Paediatrics, Diabetology and Endocrinology, Faculty of Medicine, Medical University of Gdańsk, Dębinki 7, 80-211 Gdańsk, Poland

**Keywords:** Type 1 diabetes, KL-VS polymorphism, Uric acid, Microalbuminuria

## Abstract

**Abstract:**

As the KL-VS haplotype alters secretion and activity of KLOTHO and uric acid (UA) is associated with endothelial dysfunction and inflammation, their mutual links may contribute to microalbuminuria (MA) in patients with type 1 diabetes (T1D). Therefore, we hypothesize that KL-VS polymorphism could be associated with the prevalence of MA in T1D patients, and KL-VS polymorphism could modify physiological functions and pathogenic potential of UA. We have examined 350 patients with T1D. The analysis concerned KL-VS polymorphism along with the concentrations of serum inflammatory markers, indicators of renal function, blood pressure, and lipid profile. The incidence of KL-VS genotype was lower in a group with MA in comparison to patients without this condition. Moreover, KL-VS carriers had improved indicators of renal function, lower concentrations of pro-inflammatory cytokines, and higher levels of anti-inflammatory markers. Simultaneously, among KL-VS carriers serum UA was negatively correlated with HbA1c, albumin excretion rate, ACR, CRP, TNF-α, total cholesterol, LDL-C and triglycerides, and positively correlated with HDL-C. Moreover, among wild-type *KLOTHO* carriers serum, UA was in positive correlation with creatinine, blood pressure, IL-12 and MCP-1, and in negative correlation with IL-10 and eGFR. Findings of our study suggest that the functional KL-VS polymorphism is independently associated with MA and the KL-VS genotype protects from the development of MA, and KL-VS polymorphism may modify physiological functions and pathogenic potential of UA by altering the levels of HbA1c, inflammatory biomarkers, indicators of renal function, blood pressure, and lipid profile.

**Key messages:**

• We analyzed the KL-VS polymorphism and the UA serum level in patients with T1D.

• The KL-VS polymorphism is independently associated with microalbuminuria.

• The KL-VS alleles protect from the development of microalbuminuria.

• KL-VS polymorphism may modify physiological functions and pathogenic potential of uric acid.

## Introduction

Microalbuminuria (MA), marker of endothelial dysfunction, is one of the earliest manifestations of diabetic nephropathy (DN), and its persistence is associated with higher risk of cardiovascular disease (CVD) morbidity and mortality in type 1 diabetes (T1D) patients [1]. MA serves not only as an indicator of DN risk but also as a strong predictor of its progression and is often associated with severe glomerular damage [1].

The *KLOTHO* gene has been discovered in 1997 when mice with serendipitous silencing of this gene have developed premature aging syndrome [2]. KLOTHO is highly expressed in kidneys, brain, and to a lesser extent in other organs [3]. KLOTHO exists in at least two forms, the membrane form, and the soluble secreted form. Membrane KLOTHO functions as a cofactor in fibroblast growth factor 23 signaling and as such mediates phosphate homeostasis and vitamin D metabolism [4]. The circulating form of KLOTHO functions as a hormone that exerts antioxidative, antisenescence, and antiapoptotic effects [5]. Moreover, KLOTHO deficiency causes inflammation [6], endothelial dysfunction [7], and vascular calcification [8].

The kidney is commonly recognized as the prominent tissue source of circulating KLOTHO given that it is the largest organ that expresses *KLOTHO* at high levels. Therefore, it is not surprising that patients in the early stages of chronic kidney disease already exhibit markedly reduced KLOTHO in serum [9] and urine [10] samples with the progressive decrease of the protein concentrations in more advanced stages. Moreover, KLOTHO has also been reported to have a protective effect against kidney injury in the experimental models [9].

Analyses of human *KLOTHO* polymorphisms have revealed that even slight alterations of the gene can impact both longevity and disease risk [11]. The most studied polymorphism is KL-VS, a specific haplotype in a block of six single nucleotide polymorphisms (SNPs) in a perfect linkage disequilibrium. This variant, present in approximately 15% of Caucasians, harbors two mutations in the coding region that result in amino acid substitutions and subsequent changes in the protein secretion as well as its catalytic activity [12]. Carrying one copy of the KL-VS haplotype, unlike two, has been associated with longevity and healthy cardiovascular functions [13]. On the other hand, individuals bearing two KL-VS alleles exhibited lower values of systolic blood pressure and pulse pressure in comparison to heterozygous and wild-type subjects [14].

Serum uric acid (UA) is associated with increased risk of various clinical conditions such as gout, metabolic syndrome, chronic kidney disease, diabetes mellitus, and CVD [15]. Different studies suggest that UA is a relevant and independent risk factor for developing cardiovascular and kidney diseases including diabetic nephropathy [16, 17]. UA may mediate these effects by inducing inflammation, oxidative stress, and endothelial dysfunction [18].

As KL-VS haplotype alters secretion and activity of KLOTHO [19] and UA is associated with endothelial dysfunction and inflammation, their mutual links may contribute to microalbuminuria in patients with T1D. Therefore, we hypothesize that:

1. KL-VS polymorphism could be associated with the prevalence of MA in T1D patients.
2. KL-VS polymorphism could modify physiological functions and pathogenic potential of UA.

## Materials and methods

### Subjects

The study subjects included 350 Caucasoid adolescent patients with type 1 diabetes diagnosed according to the American Diabetes Association criteria [20], recruited from the Chair and Clinics of Pediatrics, Diabetology and Endocrinology, Medical University of Gdańsk. Mean age of patients was 15.3 ± 3.5 years. Patients with coexisting autoimmune and/or inflammatory diseases were excluded from the study. All patients were treated with humanized insulin at doses of 0.87 ± 0.2 U/kg. At the time of sampling biochemical measurement of renal function, C-reactive protein (CRP) and glycated hemoglobin (HbA1c) were monitored.

The urinary albumin excretion (UAE) was expressed as the average of the three 24 h collections. Cases were classified as microalbuminuria when in at least two out of three urine samples, UAE ratio was 30-300 mg/24 h.

Written informed consent was obtained from all individuals enrolled in the study or from a parent or guardian. This study was approved by the Ethics Committee of the Medical University of Gdańsk, and the investigation was carried out in accordance with the principles of the Declaration of Helsinki.

### Medical examinations

Systolic and diastolic blood pressures (SBP and DBP, resp.) were measured using automatic 24 h ambulatory blood pressure monitoring (ABPM) by the Holter method. All the average values of the blood pressure were expressed in the centile charts.

Renal function was evaluated by estimated glomerular filtration rate (eGFR), which was estimated by using the Zappitelli equation: eGFR (ml/min/1.73 m^2^) =  (507.76 × *e*^(0.3 × height (cm))^)/(serum cystatin C (mg/l)^0.635^ × serum creatinine (μmol/l)^0.547^) [21].

### Methods

Venous blood samples were withdrawn after 12–14 h overnight fasting. Serum and plasma samples were collected from T1D patients by centrifugation at 500*g* for 15 min and stored at − 70 °C until analysis.

Concentrations of cytokines and adhesion molecules were determined using commercial enzyme-linked immunosorbent assay kits (R&D Systems, Minneapolis, MN, USA) according to the manufacturer’s protocol.

Plasma TC, TG, and HDL-C concentrations were measured in an independent, ISO certified laboratory. LDL-C was estimated by the Friedewald equation [22].

Genomic DNA from all subjects was isolated from EDTA-stabilized blood using the EXTRACTME DNA BLOOD kit (Blirt, Poland). DNA was stored at − 20 °C until the time of use. Characterization of KL-VS polymorphism was analyzed as previously described [23].

### Statistical analysis

The results were analyzed using the Statistica, ver. 12 (StatSoft, Inc., USA). Conformation of the allele frequencies to the Hardy-Weinberg equilibrium proportions was tested by the *χ*^2^ test. The genotypes and allele frequencies of the KL-VS polymorphism were compared using Fisher’s exact test. Differences between groups were analyzed by ANOVA for normally distributed values or the Kruskal–Wallis test for nonparametric values and by the *χ*^2^ Pearson test for dichotomous variables. Correlation between variables was evaluated using Spearman’s correlation coefficient. To deal with multiple testing, Benjamini-Hochberg’s correction was used for statistical significance. The level of significance was set at *p* ≤ 0.05. A multiple linear regression analysis was undertaken to determine the independence of KL-VS polymorphism and uric acid concentration from other variables. Backward multivariate regression analyses were performed with stepwise method, using *p* > 0.10 as removal criterion. Logistic regression model was used to examine the association between *KLOTHO* polymorphism and microalbuminuria.

## Results

### KLOTHO *genotype distribution*

The incidence of each genotype in *KLOTHO* gene and VS genotype frequency in T1D patients is shown in Table 1. The frequency for the VS alleles in KL-VS polymorphism was 0.124 for T1D subjects. The genotype distribution was in Hardy-Weinberg equilibrium (*p* = 0.43).

**Table 1:** KLOTHO genotype distribution in T1D patients

*KLOTHO* genotypes	T1D patients (*n* = 350)		Hardy-Weinberg equilibrium
	*n*	%	
wt/wt	270	77.1	*p* = 0.43
wt/VS	73	20.9	
VS/VS	7	2	
VS frequency	0.124		

*n*, number of patients

Hardy-Weinberg equilibrium proportions was tested by the *χ*^2^ test

### KLOTHO *genotypes and clinical characteristics of patients*

Characteristics of T1D patients included in this study differing in the KL-VS polymorphism are shown in Table 2. There were no statistically significant differences in sex, age, age of T1D onset, BMI, HbA1c, serum UA concentration, and values of blood pressure between subjects with different KL-VS genotypes. However, individuals with VS genotype had shorter duration of T1D (*p* = 0.04), higher eGFR (*p* = 0.04) and lower values of albumin excretion rate (*p* < 0.001), and albumin to creatinine ratio (ACR, *p* < 0.001) than their wild-type counterparts.

**Table 2:** KL-VS genotypes and selected clinical characteristics in T1D patients

Clinical parameter	*KLOTHO*		*p*
	wt/wt	wt/VS VS/VS	
*n* (%)	270 (77.1)	80 (22.9)	–
Sex (male/female)	153/117	41/39	0.39^*^
Age (years)	15.4 ± 3.5	15.0 ± 3.5	0.29
Age of onset of diabetes (years)	8.6 ± 3.1	8.9 ± 3.1	0.44
Duration of diabetes (years)	6.9 ± 3.3	6.1 ± 2.9	**0.04**
BMI (kg/m^2^)	19.0 ± 2.6	18.8 ± 2.7	0.40
HbA1c (%) (mmol/mol)	8.7 ± 1.8	8.6 ± 1.5	0.40
	72 ± 19	70 ± 17	
Albumin excretion rate (mg/24 h)	25 ± 18	16 ± 13	**< 0.001**
eGFR (ml/min/1.73 m^2^)	119 ± 27	126 ± 26	**0.04**
ACR (mg/g)	30 ± 21	18 ± 14	**< 0.001**
Serum uric acid (mg/dl)	3.26 ± 0.55	3.27 ± 0.49	0.89
Systolic blood pressure (mmHg)	115 ± 8	116 ± 7	0.33
Diastolic blood pressure (mmHg)	72 ± 6	73 ± 6	0.06

*n*, number of patients

Differences were calculated by the ANOVA test. Data are presented as arithmetic mean ± standard deviation (SD)

^*^χ^2^ Pearson test

*p*, Benjamini-Hochberg’s adjusted *p* value for differences between analyzed genotypes: wild-type *KLOTHO* and KL-VS alleles

Bold *p* values indicate that the differences are statistically significant

### Serum concentrations of different variables in patients with T1D differing in the KL-VS polymorphism

Table 3 describes the association between KL-VS genotypes and serum concentrations of different variables in T1D patients. There was no statistically significant difference in serum concentrations of IL-12, creatinine, total cholesterol, HDL-C, and triglycerides between subjects with different KL-VS genotypes. However, KL-VS carriers had lower concentrations of pro-inflammatory CRP (*p* = 0.001), TNF-α (*p* = 0.02), MCP-1 (*p* = 0.001), and cystatin C (*p* = 0.01). Simultaneously, the VS carriers had increased serum concentrations of anti-inflammatory IL-10 (*p* = 0.009) and LDL-C (*p =* 0.03) when compared with holders bearing wild type genotype.

**Table 3:** Serum concentrations of different variables in patients with T1D differing in the KL-VS polymorphism

Parameter	*KLOTHO*		*p*
	wt/wt	wt/VS VS/VS	
CRP (mg/l)	2.09 ± 1.85	1.41 ± 0.93	**0.001**
TNF-α (pg/ml)	1.15 ± 0.94	0.87 ± 0.98	**0.02**
IL-12 (pg/ml)	1.81 ± 2.08	1.91 ± 1.63	0.69
MCP-1 (pg/ml)	30 ± 8	27 ± 10	**0.001**
IL-10 (pg/ml)	1.57 ± 2.15	2.38 ± 3.15	**0.009**
Creatinine (mg/dl)	0.75 ± 0.12	0.73 ± 0.09	0.30
Cystatin C (mg/l)	0.63 ± 0.15	0.58 ± 0.16	**0.01**
Total cholesterol (mmol/l)	4.48 ± 0.62	4.53 ± 0.73	0.53
HDL-C (mmol/l)	1.42 ± 0.27	1.48 ± 0.23	0.08
LDL-C (mmol/l)	2.45 ± 0.54	2.66 ± 0.66	**0.003**
Triglycerides (mmol/l)	1.04 ± 0.48	0.95 ± 0.32	0.12

Differences were calculated by the ANOVA test. Data are presented as arithmetic mean ± standard deviation (SD)

*p*, Benjamini-Hochberg’s adjusted *p* value for differences between analyzed genotypes: wild-type *KLOTHO* and KL-VS alleles

Bold *p* values indicate that the differences are statistically significant

### KL-VS genotype and microalbuminuria in T1D patients

The effects of the KL-VS polymorphism on MA was evaluated by multiple linear regression analysis including significant variables: sex, inflammatory status of patients (as serum CRP concentration), duration of diabetes, uric acid concentration, and *KLOTHO* genotype (Table 4). The model revealed that KL-VS polymorphism was independently associated with MA. To clarify the effect of this polymorphism on MA, we have compared the distribution of genotypes between individuals with and without MA. Analysis of the prevalence of MA in T1D patients according to KL-VS genotypes is shown in Table 5. None deviated significantly from the Hardy-Weinberg equilibrium. Among 350 diabetic cases, 65 patients had microalbuminuria. The frequency of VS genotype was lower in patients with MA in comparison to T1D subjects without this complication (0.061 vs. 0.138; *p* = 0.006). A multivariable logistic regression analyses revealed that KL-VS polymorphism was significantly associated with MA with the minor VS genotype decreasing the risk of these conditions. The adjustment for sex, inflammatory status of patients, and duration of diabetes did not attenuate this association. The risk of MA in patient carrying VS genotype was nearly threefold lower than of non-carriers (OR = 0.359; 95% CI = 0.154-0.839; *p* = 0.02).

**Table 4:** Multiple linear regression analysis of the parameters associated with microalbuminuria in T1D patients

Covariates	*β*	*t*	*p*
Sex (male = 1)	0.180	3.504	**< 0.001**
CRP	0.158	3.022	**0.003**
Duration of diabetes	0.110	2.136	**0.03**
KL-VS polymorphism (VS = 1)	− 0.123	− 2.383	**0.04**
Uric acid	0.112	2.189	**0.03**

*R*^2^ = 0.176

Bold *p* values indicate that the differences are statistically significant

**Table 5:** Distribution of KL-VS genotype and allele frequencies in T1D patients with and without microalbuminuria

*KLOTHO* genotypes	Total subjects						
	No microalbuminuria (*n* = 285)		Microalbuminuria (*n* = 65)		Fisher’s exact test	OR	95% CI
	*n*	%	*n*	%			
wt/wt	212	74.4	58	89.2	***p*** = 0.006^*^	0.359	0.154–0.839
wt/VS	67	23.5	6	9.2			
VS/VS	6	2.1	1	1.6			
VS frequency	0.138		0.061				

*n*, number of patients

OR, odds ratio

95% CI, 95% confidence interval

^*^Significance between T1D patients with and without microalbuminuria

Bold *p* values indicate that the differences are statistically significant

### Relationship between serum uric acid concentration and different variables in T1D patients

The results of Spearman’s correlations between serum UA and different covariates are reported in Table 6.

**Table 6:** Correlation coefficients (*R*) between serum uric acid level and different variables in T1D patients

	Uric acid	
	wt/wt	wt/VS VS/VS
	***R***	
HbA1c	− 0.0736	− 0.3596^*^
Albumin excretion rate	0.0263	− 0.2338^*^
Creatinine	0.2450^*^	0.0981
eGFR	− 0.1316^*^	0.0013
ACR	0.0408	− 0.2573^*^
Systolic blood pressure	0.1250^*^	0.0189
Diastolic blood pressure	0.1375^*^	− 0.1008
CRP	0.0499	− 0.2376^*^
TNF-α	0.0685	− 0.2392^*^
IL-12	0.1558^*^	0.0711
MCP-1	0.1344^*^	0.0131
IL-10	− 0.1405^*^	− 0.0138
Total cholesterol	− 0.0228	− 0.2327^*^
HDL-C	− 0.1170	0.2374^*^
LDL-C	0.0497	− 0.2535^*^
Triglycerides	− 0.0588	− 0.2462^*^

The Spearman test was used to calculate the strength of correlation

*Benjamini-Hochberg’s adjusted *p* < 0.05 for differences between analyzed genotypes: wild-type *KLOTHO* and KL-VS alleles

Bold indicate that the differences are statistically significant

In KL-VS carriers, a significant negative correlation was observed between serum UA and HbA1c (*p* = 0.001). In contrast, UA concentration was not associated with HbA1c in the wild-type *KLOTHO* genotype patients.

In the wild-type *KLOTHO* genotype carriers serum, UA was positively correlated with the indicator of renal function such as serum creatinine concentration (*p* < 0.001) and negatively correlated with eGFR (*p* = 0.03). On the other hand, the analysis of KL-VS bearing patients revealed that UA concentration was negatively correlated with ACR (*p* = 0.02).

In the wild-type *KLOTHO* genotype carriers, a significant positive correlation was detected between serum UA concentration and systolic (*p* = 0.04) as well as diastolic (*p* = 0.02) blood pressure. In contrast, serum UA was not associated with blood pressure values in KL-VS bearing patients.

Only in KL-VS bearing patients serum UA was negatively correlated with the concentrations of inflammatory biomarkers such as CRP (*p* = 0.04) and TNF-α (*p* = 0.03). On the other hand, only in the wild-type *KLOTHO* genotype carriers serum UA was positively linked with pro-inflammatory IL-12 (*p* = 0.01) and MCP-1 (*p* = 0.03).

In the wild-type *KLOTHO* genotype carriers, a significant negative correlation has been identified between serum UA and anti-inflammatory IL-10 (*p* = 0.02). In contrast, UA concentration was not associated with IL-10 in KL-VS bearing patients.

Serum UA was in negative correlation with the concentrations of total cholesterol (*p* = 0.03), LDL-C (*p* = 0.02) and triglycerides (*p* = 0.03), and positively correlated with HDL-C (*p* = 0.04). These associations were found only in KL-VS bearing patients.

## Discussion

*KLOTHO* is a good candidate gene for the T1D due to its importance in insulin signaling and inflammation; however, our previous study [23] demonstrated that KL-VS genotype is not directly involved in genetic susceptibility to T1D. Despite this, the functional KL-VS variant of the *KLOTHO* gene protects against the development of retinopathy in patients with T1D and is likely to improve inflammatory status and delay endothelial dysfunction in these subjects [23]. In the present study, we aimed to evaluate genetic contribution of the *KLOTHO* KL-VS variant to the prevalence of MA and its potential influence on pathophysiological role of UA in T1D adolescents.

We have hypothesized that the KL-VS polymorphism could be associated with MA in T1D patients, and our results gave support to this assumption. Multiple regression analysis has shown that the KL-VS polymorphism was independently associated with MA after adjusting for some conventional risk factors. These findings indicate that *KLOTHO* genotype plays an important role in the pathogenesis of MA. We have also observed that the incidence of KL-VS genotype is lower in a group with MA in comparison to diabetic patients without this condition. The risk of MA for KL-VS genotype carriers was nearly threefold lower than for non-carriers. Moreover, KL-VS bearing patients had improved indicators of renal function such as albumin excretion rate, eGFR, ACR, and cystatin C concentration. Simultaneously, as KL-VS carriers had lower serum concentrations of pro-inflammatory markers (CRP, TNF-α, MCP-1) and higher anti-inflammatory IL-10 than non-carriers, we can assume that the KL-VS genotype is associated with weakened inflammatory response. From a clinical perspective, the presence of statistically significant differences between our data sets is of limited value, but patients with KL-VS genotype are more privileged than wild-type carriers.

There is a lot of evidence that KLOTHO is an important factor for cardiorenal health and disease [19, 24, 25]. In individuals with type 2 diabetes, the concentration of plasma soluble KLOTHO has been shown to negatively correlate with the development of albuminuria and the decline in eGFR [26]. Therefore, soluble KLOTHO concentration in plasma and urine samples may be a novel and useful marker of early diabetic renal injury [27]. Meanwhile, little is known about the role of KLOTHO in patients with T1D. In individuals with T1D, MA has been associated with soluble KLOTHO deficiency [28]. Furthermore, serum KLOTHO may have a protective effect against atherosclerosis and endothelial dysfunction in these subjects [29]. That is why further analyses are required to determine the role of KLOTHO in patients with T1D.

Many studies have explored the relationship between UA and diabetic kidney disease. It has been demonstrated that in T1D subjects, serum UA is associated with microvascular endothelial dysfunction [30], development of micro- and macroalbuminuria [31], and nephropathy [32]. High serum UA concentrations are also directly associated with the development and progression of chronic kidney disease and increased rates of kidney function loss among T1D patients [33]. UA cannot work only as an agent of endothelial dysfunction and kidney disease, but also it influences the cardiorenal etiology and risk factors. We have found such associations, depending on sex, among T1D patients [34]. In the present study, we have observed similar differences in correlations of serum UA with inflammatory biomarkers and kidney function between wild-type carriers and KL-VS bearing patients. We have shown that in subjects with KL-VS genotype serum, UA was negatively correlated with HbA1c, the indicators of renal function (albumin excretion rate, ACR), the subclinical inflammatory markers (CRP, TNF-α), and the lipid parameters (total cholesterol, LDL-C, triglycerides) as well as positively correlated with HDL-C. Moreover, in patients with wild-type *KLOTHO* genotype serum UA was positively correlated with creatinine, blood pressure, and pro-inflammatory factors (IL-12, MCP-1) as well as negatively correlated with anti-inflammatory IL-10 and eGFR. Therefore, patients with KL-VS polymorphism are probably more privileged than their wild-type counterparts.

KLOTHO is not only involved in longevity and aging, but it is also a cardiorenal protective factor which has a central impact on renal physiology and pathophysiology. Recent advances in understanding KLOTHO biology definitely support the notion that aberrant function of the protein promotes nephropathy progression and CVD development. The molecular mechanisms of KLOTHO’s reno- and cardio-protection are still being unraveled. Despite many genetic association studies on the KL-VS polymorphism, there is a limited information on the molecular mechanism behind the KL-VS association with renal physiology and pathophysiology. Given the decreased risk of MA development and inflammation, a thorough description of the alterations associated with *KLOTHO* polymorphism is important for understanding the role of KLOTHO in diabetic patients. To our knowledge, the current study for the first time provides new insights into the pathophysiological linkage between KL-VS polymorphism, serum UA, and renal function markers as well as some cardiometabolic risk factors in T1D young patients, although the detailed molecular mechanisms underlying observed *KLOTHO* genotype differences remain to be explored.

The present study has several strengths and limitations that need to be addressed briefly. The strengths include the use of a pure Caucasoid population from the north region of Poland to eliminate false positive results due to population stratification. Moreover, all of the enrolled patients had relatively preserved renal function (eGFR > 70), a similar duration of diabetes, and comprised well-characterized cohort attending a single center for their diabetes care. Regarding limitations, firstly, although the study cohort is homogenous and well-characterized, it may be considered relatively small. Secondly, despite having detected *KLOTHO* genotype-based differences in the correlations between UA concentration and inflammatory biomarkers as well as kidney function, there are still many unmeasured genetic and environmental factors that influence these associations. Thirdly, our statistical power to assess the correlation of serum UA and measured values was limited. Finally, we have not measured serum and urinary KLOTHO concentrations—it is an impediment to interpret observed relationships and their biological significance. In spite of these limitations, our findings emphasize the role of KL-VS polymorphism in patients with T1D.

In conclusion, findings of our study suggest that:

1. The functional KL-VS polymorphism is independently associated with MA in such manner that the KL-VS genotype protects against the development of MA in adolescents with T1D.
2. KL-VS polymorphism may modify physiological functions and pathogenic potential of UA by altering the levels of HbA1c, inflammatory biomarkers, indicators of renal function, blood pressure, and lipid profile.
